# Influence of Radical Generation and Elimination on Sebum Production of Hamster Sebaceous Gland Cells

**DOI:** 10.3390/life15020165

**Published:** 2025-01-24

**Authors:** Yoshihiro Tokudome, Asami Sasaki

**Affiliations:** 1 Laboratory of Cosmetic Sciences, Institute of Ocean Energy, Saga University, 1 Honjo, Saga 840-8502, Japan; 2 Laboratory of Cosmetic Sciences, Graduate School of Science and Engineering and Advanced Health Sciences, Saga University, 1 Honjo, Saga 840-8502, Japan; 3 Laboratory of Dermatological Physiology, Faculty of Pharmacy and Pharmaceutical Sciences, Josai University, 1-1 Keyakidai, Sakado 350-0295, Japan

**Keywords:** antioxidant, cell differentiation, oxidant, sebum production

## Abstract

We focused on the effects of radical induction on cell differentiation and sebum production when antioxidants and oxidants were applied to normal hamster sebaceous gland cells. We also examined the relationship between sebum production and the reactive oxygen species (ROS) scavenging rate in these cells. Eight antioxidants (fullerene, epigallocatechin gallate, α-glucosylrutin, copper (II) gluconate, tannic acid, sodium copper-chlorophyllin, phytic acid, and ascorbyl tocopheryl phosphate) and one oxidant (hydrogen peroxide, H_2_O_2_) were used. The number of differentiated cells was determined by counting the viable cells, the intracellular triglyceride (TG) level was determined by separation and quantification by HPTLC, and the superoxide anion radical scavenging rate, nitric oxide scavenging rate, and H_2_O_2_ scavenging rate were also investigated. Adding various antioxidants decreased the differentiated cell number and TG content in the hamster sebaceous gland cells. Meanwhile, adding an oxidant (H_2_O_2_) increased the differentiated cell number and cellular TG. Pretreatment with antioxidants also prevented the oxidants from increasing the differentiated cell number and TG level. A strong correlation between the intracellular TG content and the H_2_O_2_ scavenging rate was identified. These results indicate that radical generation and scavenging are involved in sebum production in hamster sebaceous gland cells, and that the scavenging rate of H_2_O_2_ may be particularly important.

## 1. Introduction

The sebaceous gland opens into the upper part of the hair follicle of the dermis layer and is composed of sebaceous gland cells. These cells form intracellular lipid droplets upon differentiation [1]. Finally, the cells themselves burst, and sebum is secreted onto the skin’s surface [2]. The main component of sebum is triglycerides (TGs), constituting approximately 45% of the total, along with approximately 20% free fatty acids (FFAs) and 5% cholesterol [3]. The TG secreted from sebaceous gland cells is degraded by the lipase produced by Propionibacterium acnes and FFAs are also produced during this process [3]. Cholesterol is considered to be a remnant of components such as the cell membrane that is generated when sebaceous gland cells disintegrate [3]. In humans, sebaceous glands are distributed throughout the skin, except for on the palms of hands, the soles of the feet, the face, and the back. The size and morphology of these glands vary greatly with age and sex, and sebum secretion is stimulated by endogenous factors such as hormones and by exogenous factors such as ultraviolet radiation [4]. In particular, sebaceous gland development and sebum secretion are markedly altered by the actions of male hormones [5]. Testosterone, the main male hormone present in blood, is thought to promote sebaceous gland enlargement [6], cell division [7], and sebum production [8,9], which, as a result, enhances sebum secretion. Sebum production is closely linked to skin conditions. For example, too much sebum can lead to the development of acne and comedones. Meanwhile, the conditions associated with too little sebum include atopic dermatitis and senile xerosis. Researchers interested in clinical practices are therefore investigating ways to control sebum secretion.

Sebum production is regulated by several factors. The substances that stimulate sebum secretion include 5α-dihydrotestosterone (5α-DHT) [5], the adrenocorticotropin-releasing hormone (CRH) [10], insulin [11] and the insulin-like growth factor 1 (IGF-1) [12], peroxisome proliferator-activated receptor γ (PPARγ) agonists [13,14], and lipid mediators such as prostaglandin J2 (PGJ2) [15] and leukotriene B4 (LTB4) [16]. In recent years, it has been reported that sebum secretion is also regulated by several factors such as inflammatory mediators, neurotransmitters, and endocrine signals [17]. Moreover, it has been reported that fullerene, known as an antioxidant, suppresses sebum production [18]. Meanwhile, female hormones [19], retinoic acid [20,21,22], the epidermal growth factor (EGF) [23,24], and other factors inhibit sebum production. Recently, piperine has been reported to suppress sebum production by downregulating PPARγ [25].

In this paper, we focus on the effects of radical induction on cell differentiation and sebum production when antioxidants and oxidants are applied to normal hamster sebaceous gland cells in order to elucidate the basic action of sebaceous gland cells.

## 2. Materials and Methods

### 2.1. Matrerials

#### Samples and Reagents

The hydrogen peroxide (H_2_O_2_), α-glucosylrutin, copper (II) gluconate, tannic acid, sodium copper chlorophyllin, and 50% phytic acid solution were purchased from Fuji Firm Wako Pure Chemical Industries. The epigallocatechin gallate was purchased from LKT Laboratories (St. Paul, MN, USA). The fullerene was obtained from BioResearch Co., Ltd. (Tokyo, Japan). The ascorbyl tocopheryl phosphate (EPC) was provided by Senju Pharmaceutical (Osaka, Japan).

The phosphate-buffered saline (PBS) was purchased from Takara Bio Inc. (Otsu, Japan). The chloroform, methanol, hexane, diethyl ether, acetic acid, copper sulfate, palmitic acid, cholesterol, sulfanilamide, N-1-naphthylethylenediamine dihydrochloride, phosphoric acid, sodium nitrite, horseradish peroxidase (HRP), boric acid, and luminol were obtained from Fuji Firm Wako Pure Chemical Industries, Ltd. (Osaka, Japan). The triolein, nitrotetrazolium blue chloride (NBT), hypoxanthine, and xanthine oxidase (XOD) were purchased from Sigma Aldrich (St. Louis, MO, USA). Amplex Red (10-acetyl-3,7-dihydroxyphenoxazine: ADHP) was purchased from Cayman Chemical Company (Ann Arbor, MI, USA).

### 2.2. Cell and Cell Culture

The normal hamster sebaceous gland cells derived from golden hamsters were purchased from Kurabo Co., Ltd. (Osaka, Japan). A Humedia-BG medium for sebocyte proliferation and Humedia-BD for inducing the differentiation of sebocytes were purchased from Kurabo Co., Ltd. (Osaka, Japan).

The normal hamster sebaceous gland cells were seeded in a 24-well plate at 1.0 × 10^4^ cells/well and cultured under conditions of 37 °C and 5% CO_2_. Humedia-BG, a medium for sebaceous gland cell growth supplemented with growth additives (8% fetal bovine serum: FBS, 2% human serum: HS), was used to culture the solution and 10 μg/mL of the human recombinant epidermal growth factor (hEGF) was replaced every other day for 5 days.

### 2.3. Sample Addition

After five days of culture, the culture medium was changed to Humedia-BD, a medium for inducing sebaceous gland cell differentiation with differentiation-inducing additives (FBS, HS, 3 μg/mL of insulin). The control group was treated with the medium only, and the sample-applied group was prepared with the medium and samples were added every other day for 10 days. Various antioxidants were added at concentrations of 0.001 to 5 mM.

In the radical induction experiment, the samples were changed and cultured in the first half of day 10 and in the second half of days 6 to 10. The antioxidant used was 0.1 mM of phytic acid, which was highly effective in various studies, and the oxidant was 100 μM of H_2_O_2_ (Table 1).

### 2.4. Viability of Cells

The cells were collected on the 10th day after the addition of the sample. Cell viability was measured using the Trypan Blue method and cell counting.

### 2.5. Extraction of Lipids from Cells

After 10 days of sample addition, the medium was removed, and the cells were washed twice with PBS. The cells were collected in a Falcon tube using a cell scraper and washed again with 0.3 mL of purified water. Chloroform (1 mL) and methanol (2 mL) were added and stirred so that the ratio of chloroform–methanol–purified water was 1:2:0.8 (*v*/*v*%). The cells were sonicated using an ultrasonic homogenizer (Advanced Sonifier 250; Branson Ultrasonics, Danbury, CT, USA) under the conditions of output control 2 and duty cycle 50% for 10 s, and the cell residue was filtered using a glass syringe and a filter (Millex GN 0.20 µm; Millipore, Billerica, MA, USA). Then, 1.5 mL of chloroform, 0.5 mL of methanol, and 0.65 mL of purified water were added to the tube before filtration so that the final ratio after filtration was chloroform–methanol–purified water = 2:2:1.8 (*v*/*v*%). After stirring, the mixture was centrifuged (Himac CT 6E; Hitachi, Tokyo, Japan) at room temperature and 130× *g* for 20 min and separated into two layers. The upper layer (water–methanol layer) of the solution was removed and the lower layer containing the lipid (chloroform layer) was evaporated to dryness by nitrogen streaming using a drive lock bath (Dry Thermo Unit DTU-IC; Taitec, Koshigaya, Japan). The lipid extraction process was carried out by partly modifying the Bligh–Dyer method [26].

### 2.6. Quantitative Analysis of Lipids

The dried sample was re-dissolved with 100 µL of a solution of chloroform–methanol at a 2:1 ratio and HPTLC (HPTLC Silica Gel 60; Merck, Darmstadt, Germany) was performed using a capillary (Ringcaps; Hirschman Laborgeräte, Eberstadt, Germany). In addition, 10 μL of triglyceride (concentration: 10, 20, 50, 100, 200 and 500 µg/mL) was spotted as a standard. HPTLC was developed twice using hexane–diethylether–acetic acid at a ratio of 80:20:1 (*v*/*v*%). In addition, 8% of a phosphoric acid aqueous solution containing 10% copper(II) sulfate pentahydrate was sprayed on the HPTLC and heated using the TLC heater III (CAMAG, Muttenz, Switzerland) at 180 °C for 10 min. The obtained HPTLC plate was imaged with a Lumino image analyzer (LAS-1000 Plus; FUJIFILM Corporation, Tokyo, Japan) and the intracellular triglyceride level was quantified using a Multi Gauge (Fuji Film Co., Tokyo, Japan).

### 2.7. Measurement of Differentiated Cell Number

After 10 days of sample addition, the medium was removed, and the cells were washed twice with PBS. The cells were observed using an inverted system microscope (IX71; Olympus, Tokyo, Japan) and analyzed by DP2-BSW (Olympus). The number of differentiated cells was measured and, after correction with the area (cm^2^), the number of differentiated cells per area was calculated.

### 2.8. Determination of Scavenging Efficiency

#### 2.8.1. Superoxide Anion Radical

The superoxide anion radical scavenging efficiency of various antioxidants was determined by the NBT reduction method [27,28]. Briefly, a total of 100 μL of a reaction buffer containing 0.25 mM of NBT, 1.0 mM of hypoxanthine, 0.1 mM of EDTA dissolved in PBS, 25 μL of the sample, and 25 μL of 2 units/mL XOD solution prepared with 1 mg/mL of a BSA solution was added to a 96-well plate, mixed, and incubated at 37 °C for 10 min. The absorbance of formazan at a wavelength of 550 nm was measured using a microplate reader (SpectraMax M2; Molecular Devices, Silicon Valley, CA, USA). The superoxide anion radical scavenging efficiency was calculated using Equation (1).

(1) $$Superoxide\;anion\;radical\;scavenging\;efficiency\left(\%\right)=1-\left(a-c\right)÷b\times 100$$

where (*a*) is the sample + XOD solution, (*b*) is the PBS + XOD solution, and (*c*) is the sample + PBS.

#### 2.8.2. Nitric Oxide

The nitric oxide scavenging efficiency of various antioxidants was measured by the Griess reaction [29]. Briefly, a total of 50 μL of a Griess reagent, dissolving 2% sulfanilamide, 0.2% naphthylethylenediamine dihydrochloride, and 4% phosphoric acid in a PBS, and 50 μL of the sample were added to a 96-well plate, mixed, and kept for 5 min. Then, 50 μL of 1 mM sodium nitrite was added and incubated at 37 °C for 10 min. The absorbance of the azo dye at a wavelength of 540 nm was measured using a microplate reader. The nitric oxide scavenging efficiency was calculated using Equation (2).

(2) $$Nitric\;oxide\;scavenging\;efficiency\left(\%\right)=1-\left(d-f\right)÷e\times 100$$

where (*d*) is the sample + Griess solution, (*e*) is the PBS + Griess solution, and (*f*) is the sample + PBS.

#### 2.8.3. Hydrogen Peroxide (H_2_O_2_)

The H_2_O_2_ scavenging efficiency of various antioxidants was determined by an Amplex Red/HRP coupled method [30,31]. Briefly, a total of 100 μL of 400 μM Amplex Red [28] dissolved in a solution of PBS:DMSO at a ratio of 1:5 (*v*/*v*%), 50 μL of the sample, and 50 μL of a mixture of 16 mM H_2_O_2_ and 2 U/mL HRP were added to a 96-well plate and incubated at room temperature for 30 min. The absorbance of resorufin was measured at a wavelength of 480 nm using a microplate reader. The H_2_O_2_ scavenging efficiency was calculated using Equation (3).

(3) $$H_{2}O_{2}\;scavenging\;efficiency\left(\%\right)=1-\left(g-i\right)÷h\times 100$$

where (*g*) is the sample + Amplex red solution, (*h*) is the PBS + Amplex red solution, and (*i*) is the sample + PBS.

### 2.9. Statistical Analysis

The data are presented as the mean ± standard deviation. A statistical analysis was performed by Dunnett’s or Tukey’s multiple comparison test (using SAS software version 9.2, SAS Institute, Cary, NC, USA).

## 3. Results

### 3.1. Effect of Antioxidants on Viability of Hamster Sebaceous Gland Cells

**Figure 1:**
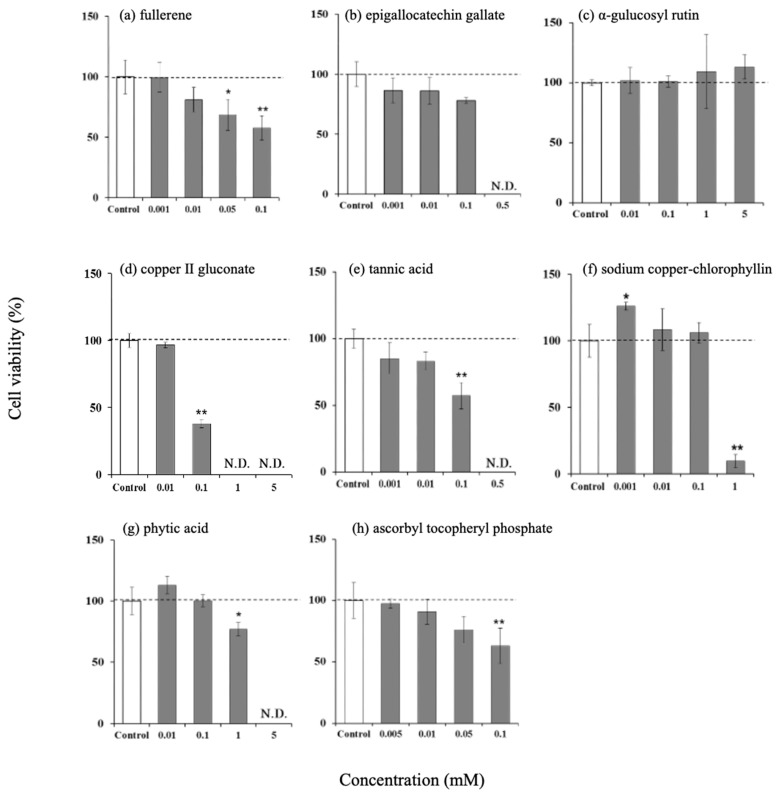
Effect of various antioxidants on cell viability in hamster sebocytes. (**a**) Fullerene, (**b**) epigallocatechin gallate, (**c**) α-glucosylrutin, (**d**) copper (II) gluconate, (**e**) tannic acid, (**f**) sodium copper-chlorophyllin, (**g**) phytic acid, and (**h**) ascorbyl tocopheryl phosphate. Values were the mean ± SD of three experiments. N.D.: Not detected, * *p* < 0.05, and ** *p* < 0.01 (versus control), according to Dunnett’s test.

To investigate the concentration at which cytotoxicity occurred, various antioxidants were applied to the hamster sebaceous gland cells, after culturing these cells. Cell viability after the addition of 0.01 mM fullerene, 0.1 mM epigallocatechin gallate, 5 mM α-glucosylrutin, 0.01 mM copper (II) gluconate, 0.01 mM tannic acid, 0.1 mM sodium copper chlorophyllin, 0.1 mM phytic acid, and 0.05 mM EPC was not significantly different from that in the control group (Figure 1). The concentrations that showed no significant differences were considered not cytotoxic and used in subsequent experiments.

### 3.2. Effect of Antioxidants on the Number of Differentiated Cells in Hamster Sebaceous Gland Cells

**Figure 2:**
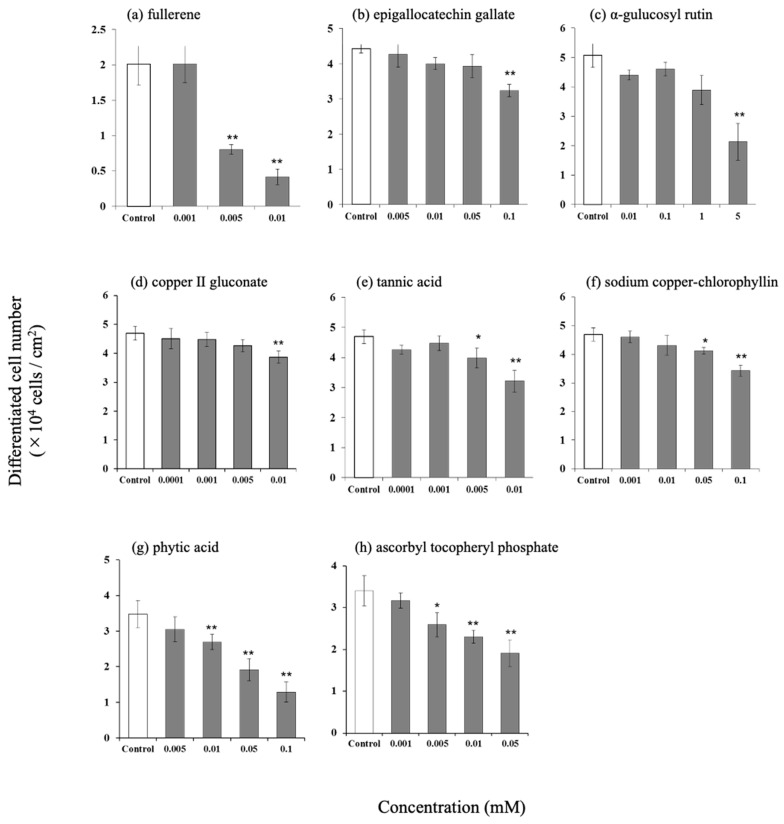
Effect of various antioxidants on differentiated cell number in hamster sebocytes. (**a**) Fullerene, (**b**) epigallocatechin gallate, (**c**) α-glucosylrutin, (**d**) copper (II) gluconate, (**e**) tannic acid, (**f**) sodium copper-chlorophyllin, (**g**) phytic acid, and (**h**) ascorbyl tocopheryl phosphate. Values are the mean ± SD of three experiments, * *p* < 0.05, and ** *p* < 0.01 (versus control), according to Dunnett’s test.

The number of differentiated cells decreased in a concentration-dependent manner upon the addition of fullerene, epigallocatechin gallate, α-glucosylrutin, copper (II) gluconate, tannin acid, sodium copper chlorophyllin, phytic acid, and EPC (Figure 2).

### 3.3. Effect of Antioxidants on Intracellular Triglyceride Content in Hamster Sebaceous Gland Cells

**Figure 3:**
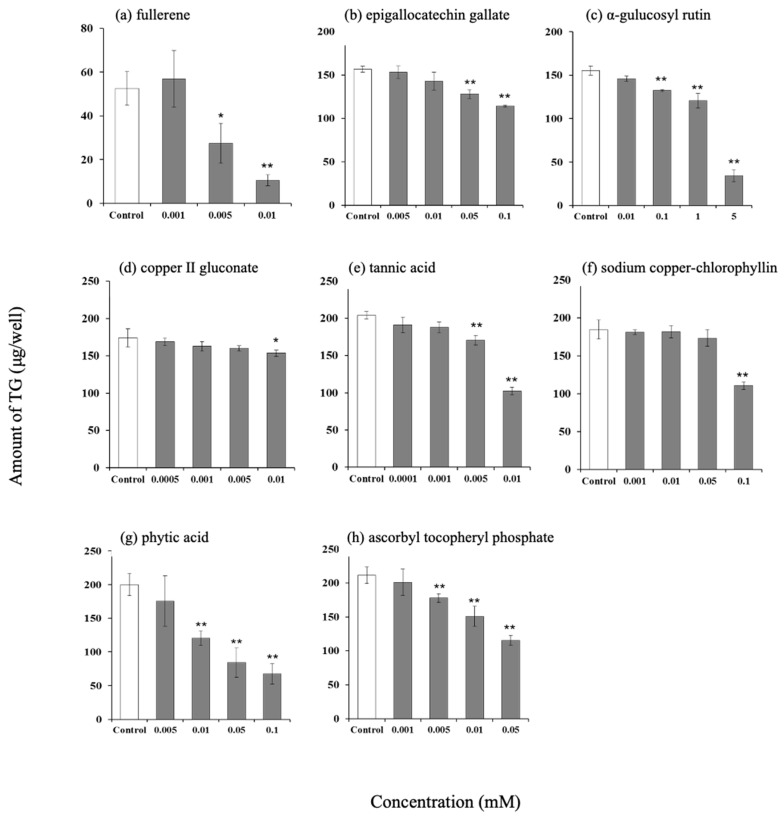
Effect of various antioxidants on amount of triglyceride in hamster sebocytes. (**a**) Fullerene, (**b**) epigallocatechin gallate, (**c**) α-glucosylrutin, (**d**) copper (II) gluconate, (**e**) tannic acid, (**f**) sodium copper-chlorophyllin, (**g**) phytic acid, and (**h**) ascorbyl tocopheryl phosphate. Values are the mean ± SD of three experiments, * *p* < 0.05, and ** *p* < 0.01 (versus control), according to Dunnett’s test.

The intracellular TG levels decreased in a concentration-dependent manner after the addition of fullerene, epigallocatechin gallate, α-glucosylrutin, copper (II) gluconate, tannin acid, sodium copper chlorophyllin, phytic acid, and EPC (Figure 3).

### 3.4. Effect of H_2_O_2_ on Viability of Hamster Sebaceous Gland Cells

**Figure 4:**
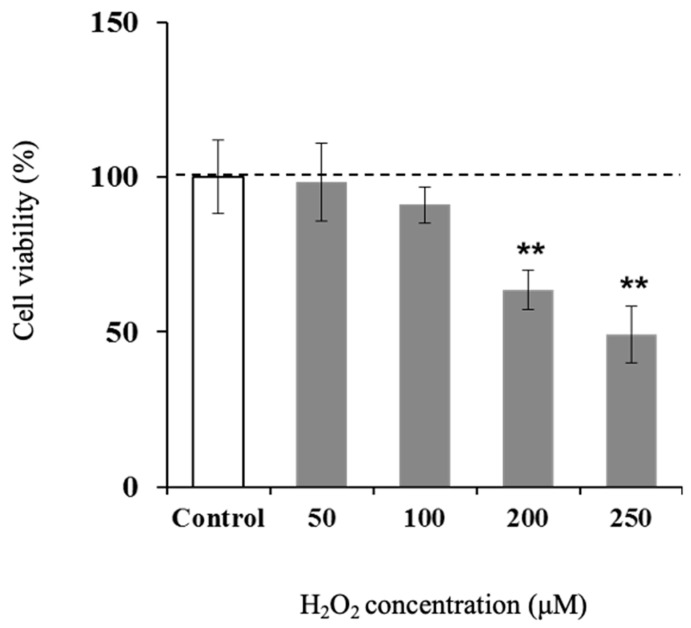
Effect on cell viability of H_2_O_2_ in hamster sebocytes. Values are the mean ± SD of three experiments, and ** *p* < 0.01 versus control, according to Dunnett’s test.

There was no significant difference in cell viability at the concentrations of H_2_O_2_ below 100 μM when compared to the control group (Figure 4). The concentration at which no significant difference was observed was defined as the non-toxic concentration, and concentrations below 100 μM were used in the subsequent experiments.

### 3.5. Effect of H_2_O_2_ on the Number of Differentiated Cells and Intracellular Triglyceride Content of Hamster Sebaceous Gland Cells

**Figure 5:**
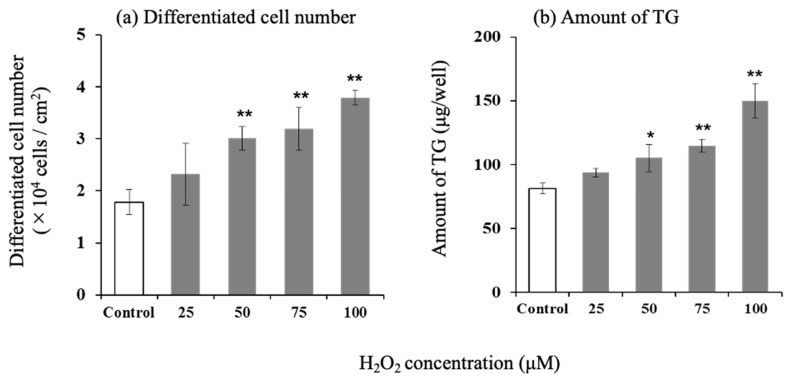
Effect of H_2_O_2_ on differentiated cell number and amount of triglyceride in hamster sebocytes. (**a**) Differentiated cell number and (**b**) amount of TG. Values are the mean ± SD of three experiments, * *p* < 0.05, and ** *p* < 0.01 versus control, according to Dunnett’s test.

The number of differentiated cells and the amount of intracellular TG increased in a concentration-dependent manner with the addition of H_2_O_2_ (Figure 5).

### 3.6. Effect of Radical Induction on the Number of Differentiated Cells and Intracellular Triglyceride Content of Hamster Sebaceous Gland Cells

**Figure 6:**
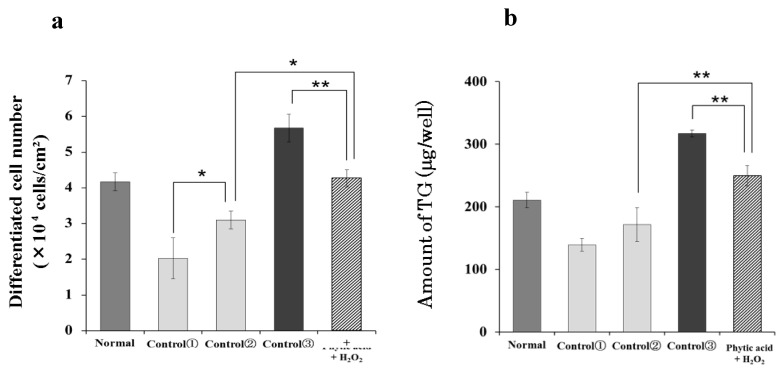
Effect of induced radicals on differentiated cell number and amount of triglyceride in hamster sebocytes. (**a**) Differentiated cell number (×10^4^ cells/cm^2^), (**b**) Amount of TG (μg/well). Values are the mean ± SD of three experiments, * *p* < 0.05, and ** *p* < 0.01, according to Tukey’s post hoc multiple comparison test.

Samples were added every other day for 10 days according to the sample addition schedule in Table 1. The number of differentiated cells and the amount of intracellular TG were measured. Compared to those in the normal group, the number of differentiated cells and the amount of intracellular TG decreased in the control (1) and (2) groups to which phytic acid was applied. The intracellular TGs and the number of differentiated cells decreased as the application time of phytic acid increased. In the control (3) group, in which H_2_O_2_ was applied, the number of differentiated cells increased when compared with that in the normal group. In the phytic acid + H_2_O_2_ group, sebum secretion increased more than in the control (2) group, which had phytic acid applied in the first half of the treatment period, and it was suppressed more than in the control (3) group, which had H_2_O_2_ applied in the second half of the treatment period (Figure 6).

### 3.7. Efficiency of Various Antioxidants

#### 3.7.1. Superoxide Anion Radical Scavenging

**Figure 7:**
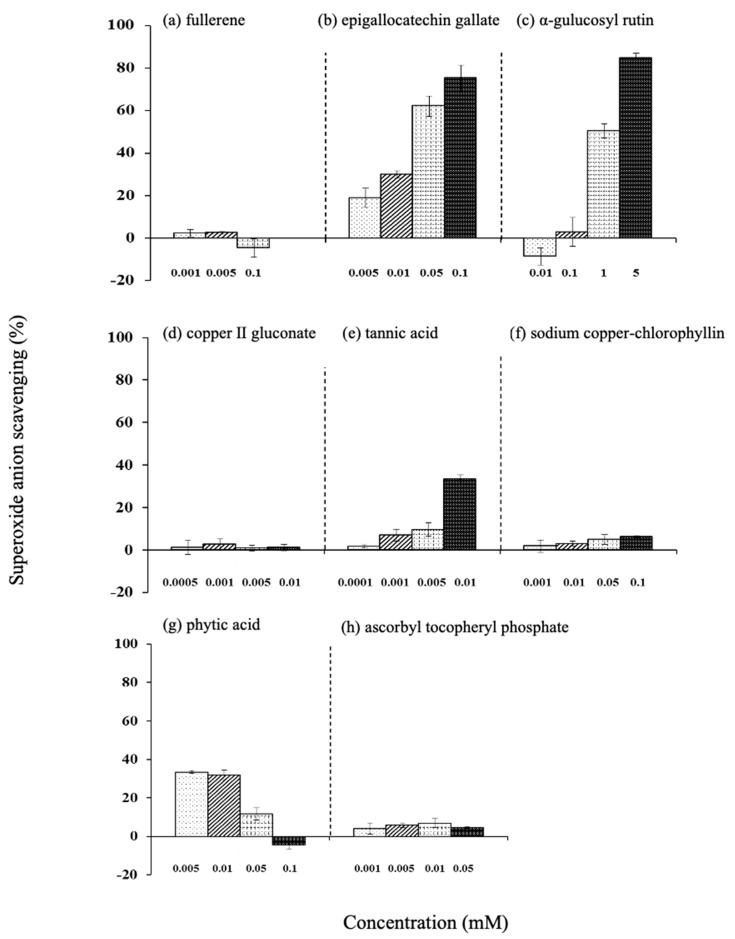
Effect of various antioxidants on superoxide scavenging. (**a**) Fullerene, (**b**) epigallocatechin gallate, (**c**) α-glucosylrutin, (**d**) copper (II) gluconate, (**e**) tannic acid, (**f**) sodium copper-chlorophyllin, (**g**) phytic acid, and (**h**) ascorbyl tocopheryl phosphate. Values are the mean ± SD of three experiments.

The superoxide anion radical scavenging efficiency of epigallocatechin gallate, α-glucosylrutin, tannic acid, and sodium copper chlorophyllin increased in a concentration-dependent manner. Among them, epigallocatechin gallate and α-glucosylrutin showed high values (Figure 7).

#### 3.7.2. Nitric Oxide Scavenging Efficiency

**Figure 8:**
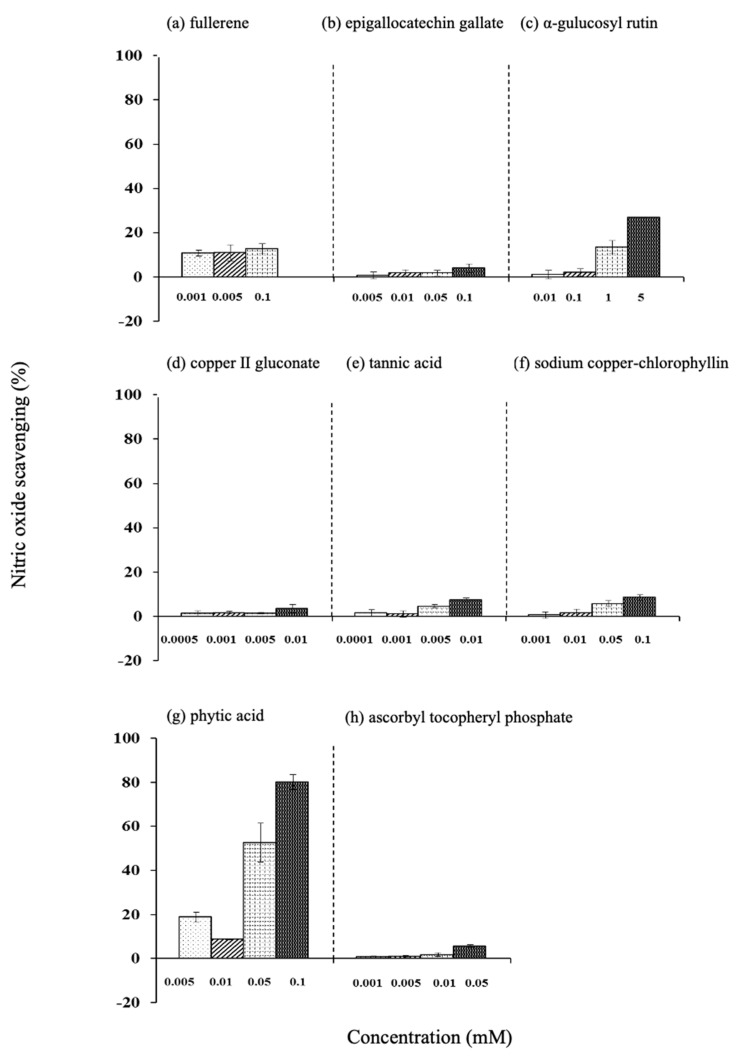
Effect of various antioxidants on nitric oxide scavenging. (**a**) Fullerene, (**b**) epigallocatechin gallate, (**c**) α-glucosylrutin, (**d**) copper (II) gluconate, (**e**) tannic acid, (**f**) sodium copper-chlorophyllin, (**g**) phytic acid, and (**h**) ascorbyl tocopheryl phosphate. Values are the mean ± SD of three experiments.

The nitric oxide scavenging efficiency of epigallocatechin gallate, α-glucosylrutin, phytic acid, and EPC increased in a concentration-dependent manner. Among them, phytic acid showed a high value (Figure 8).

#### 3.7.3. H_2_O_2_ Scavenging Efficiency

**Figure 9:**
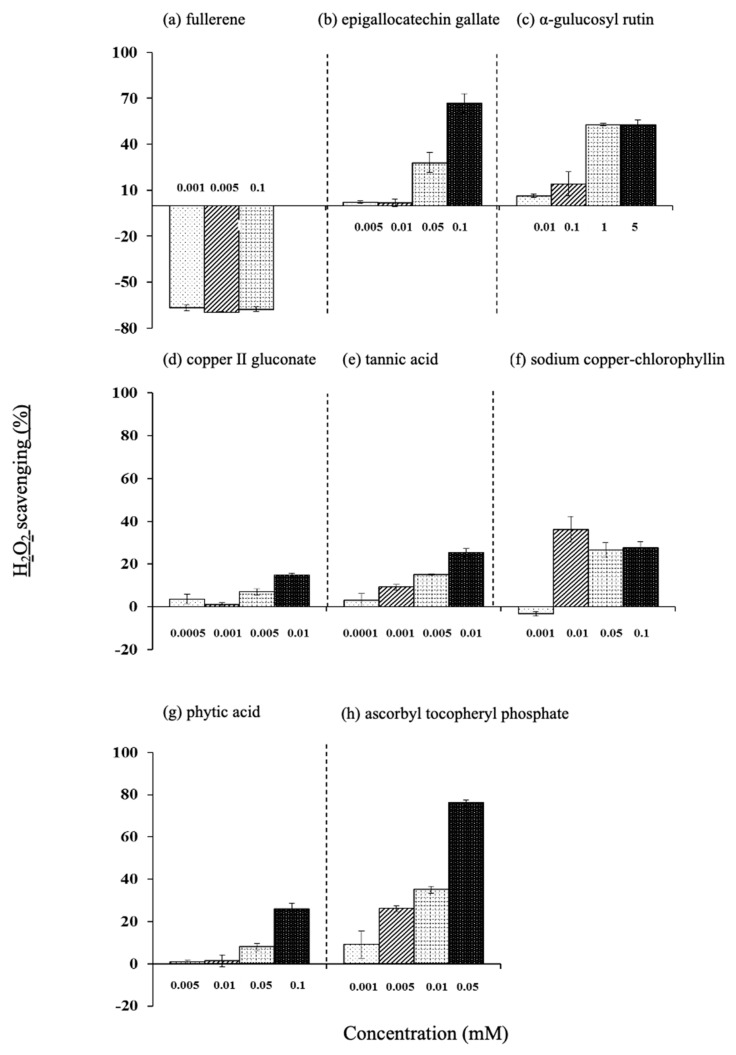
Effect of various antioxidants on H_2_O_2_ scavenging ability. (**a**) Fullerene, (**b**) epigallocatechin gallate, (**c**) α-glucosylrutin, (**d**) copper (II) gluconate, (**e**) tannic acid, (**f**) sodium copper-chlorophyllin, (**g**) phytic acid, and (**h**) ascorbyl tocopheryl phosphate. Values are the mean ± SD of three experiments.

The H_2_O_2_ scavenging efficiency of epigallocatechin gallate, tannic acid, phytic acid, and EPC increased in a concentration-dependent manner. Among them, epigallocatechin gallate and EPC showed high values (Figure 9).

### 3.8. Correlation Between Sebum Production and Reactive Oxygen Species (ROS) Scavenging Efficiency in Hamster Sebaceous Gland Cells

**Figure 10:**
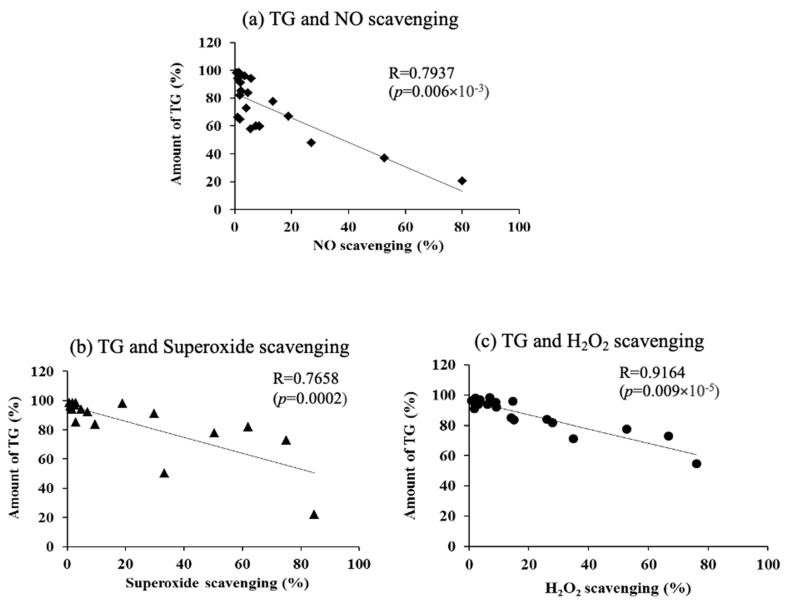
The relationship between triglyceride amount and reactive oxygen species scavenging ability. (**a**) Relationship between amount of TG and NO scavenging, (**b**) relationship between amount of TG and superoxide scavenging, and (**c**) relationship between amount of TG and H_2_O_2_ scavenging. Values are the mean of three experiments, according to Pearson’s test.

The relationship between the inhibitory effect of various antioxidants on sebum production and ROS scavenging capacity (nitric oxide scavenging capacity (Figure 10a), superoxide anion radical scavenging capacity (Figure 10b), and H_2_O_2_ scavenging capacity (Figure 10c)) is shown.

The intracellular TG levels decreased with an increasing ROS scavenging capacity. Relatively high correlations were obtained in all cases. Among them, the highest correlation was observed between the amount of intracellular TGs and H_2_O_2_ scavenging efficiency (Figure 10).

## 4. Discussion

Sebaceous gland cells form lipid droplets within cells as they differentiate, and it has been reported that sebum is eventually secreted onto the skin’s surface by a rupture of the cells themselves [1]. It has been shown that the addition of certain antioxidants to hamster sebaceous glands suppresses cell differentiation and reduces sebum content [18,32]. Based on these reports, we hypothesized that the addition of oxidants to the sebaceous glands of hamsters promotes cell differentiation and increases sebum secretion; that is, the generation and scavenging of radicals may be involved in sebum secretion. Therefore, in this study, we investigated the effects of radical production and scavenging by antioxidants and oxidants on the inhibition of sebum production, and examined the correlation between the scavenging capacity of the various radicals of antioxidants and sebum production. In addition, the hamster sebaceous gland cells were used for the following reasons. Such cells are considered a useful alternative to human sebaceous gland cells in sebaceous gland research because (1) they can be passaged and cultured for long periods and (2) their characteristics are similar to those of human sebaceous gland cells.

First, the effects of eight antioxidants (fullerene, epigallocatechin gallate, β-glucosylrutin, copper (II) gluconate, tannic acid, sodium copper chlorophyllin, phytic acid, and EPC) and an oxidant (H_2_O_2_) on the number of differentiated cells and intracellular lipid levels in hamster sebaceous gland cells were examined. The intracellular lipids in these cells comprise approximately 45% TGs and contribute most to lipid synthesis. Therefore, in this study, the TG content was measured as an indicator of intracellular lipid content. The addition of various antioxidants decreased the number of differentiated cells and intracellular TG content in a concentration-dependent manner (Figure 6 and Figure 7). In contrast, the addition of an oxidant (H_2_O_2_) increased the number of differentiated cells and intracellular TG content in a concentration-dependent manner (Figure 9). Meanwhile, even though the addition of phytic acid, an antioxidant, suppressed sebum secretion, the addition of H_2_O_2_, an oxidant, stimulated it (Figure 10). It has been reported that perilipin localized on the surface of sebaceous droplets is involved in sebum accumulation [33]. Insulin and PPARγ agonists, which are sebum production stimulators, promote the production of perilipin [34]. Meanwhile, perilipin is known to be a phosphorylated protein in adipocytes, which, like sebaceous gland cells, form intracellular fat droplets [35]. The phosphorylation of perilipin inhibits the formation of fat droplets. The UVB irradiation of differentiated hamster adipocytes was shown to cause intracellular fat droplet miniaturization and promote perilipin phosphorylation [36]. It has been reported that an active sterol regulatory element binding protein (SREBP) regulates fatty acids [37]. It has also been reported that perilipin may regulate SREBP [38]. It is possible that perilipin and SREBP-1 are related to reactive oxygen species and redox reactions, but the details are unknown and will be the subject of future research.

It has been reported that mTORC 1 (mammalian target of rapamycin complex 1) is involved in the regulation of sebaceous gland size as a mechanism for controlling sebum production [32]. It has also been reported that the activity of mTORC1 in the cytoplasm is promoted by an increase in ROS [39]. These reports suggest that the generation of ROS in cells promotes the activity of mTORC1, which in turn increases the size of sebaceous gland cells and ultimately enhances sebum secretion. The results also suggest that the addition of antioxidants or oxidants may increase or decrease ROS in sebaceous gland cells and regulate sebum secretion via mTORC1. However, the details of the mechanism involved need to be investigated in future work. Next, to confirm that radical scavenging is involved in the suppression of sebum secretion, we measured three reactive oxygen species scavenging rates (A, B, and C) using various antioxidants. We also examined the correlation between them and intracellular TG levels in hamster sebaceous gland cells. The results showed a high correlation with sebum content for all the ROS scavenging rates. Among them, the highest correlation was obtained with the H_2_O_2_ removal rate (Figure 10c). Mitochondria are known as a source of ROS [40]. The H_2_O_2_ produced in mitochondria is amphiphilic and thus highly membrane-permeable; it is considered to be relatively stable among the reactive oxygen species and presents at high concentrations in cells. Therefore, the most important contribution to the oxidative action of H_2_O_2_ was found to be the reducing action of antioxidants. These results suggest that, in sebaceous gland cells, there is a correlation between the ROS scavenging ability of antioxidants and the amount of intracellular TGs, with a particularly high correlation between H_2_O_2_ scavenging ability and the amount of sebum. In future work, it will be necessary to examine whether similar results can be obtained in human sebaceous gland cells and in vitro, and to quantitatively examine the degree of the differentiation of sebaceous gland cells by Western blotting and PCR in order to investigate how such differentiation is inhibited.

## 5. Conclusions

In this study, we found that the radical scavenging and radical generating activities of antioxidants and oxidants affect the cell differentiation of hamster sebaceous gland cells and regulate sebum production. We have also shown that hydrogen peroxide is a major contributor to sebum production among the reactive oxygen species. This suggests that the generation and scavenging of radicals may be useful in controlling sebum levels, and it is hoped that this will be applied to cosmetics and pharmaceuticals.

## Figures and Tables

**Table 1:** Radical induced experimental groups upon 10 days of treatment with phytic acid and H_2_O_2_.

Group	1–5 Days	6–10 Days
Normal	None	None
Control (1)	0.1 mM phytic acid	0.1 mM phytic acid
Control (2)	0.1 mM phytic acid	None
Control (3)	None	H_2_O_2_
Antioxidant + H_2_O_2_	0.1 mM phytic acid	H_2_O_2_

## Data Availability

The data presented in this study are available on request from the corresponding author.
